# Molluscum contagiosum virus MC80 sabotages MHC-I antigen presentation by targeting tapasin for ER-associated degradation

**DOI:** 10.1371/journal.ppat.1007711

**Published:** 2019-04-29

**Authors:** Ian B. Harvey, Xiaoli Wang, Daved H. Fremont

**Affiliations:** 1 Department of Pathology & Immunology, Washington University School of Medicine, St. Louis, Missouri, United States of America; 2 Department of Biochemistry & Molecular Biophysics, Washington University School of Medicine, St. Louis, Missouri, United States of America; 3 Department of Molecular Microbiology, Washington University School of Medicine, St. Louis, Missouri, United States of America; National Institute of Allergy and Infectious Diseases, National Institutes of Health, UNITED STATES

## Abstract

The human specific poxvirus molluscum contagiosum virus (MCV) produces skin lesions that can persist with minimal inflammation, suggesting that the virus has developed robust immune evasion strategies. However, investigations into the underlying mechanisms of MCV pathogenesis have been hindered by the lack of a model system to propagate the virus. Herein we demonstrate that MCV-encoded MC80 can disrupt MHC-I antigen presentation in human and mouse cells. MC80 shares moderate sequence-similarity with MHC-I and we find that it associates with components of the peptide-loading complex. Expression of MC80 results in ER-retention of host MHC-I and thereby reduced cell surface presentation. MC80 accomplishes this by engaging tapasin via its luminal domain, targeting it for ubiquitination and ER-associated degradation in a process dependent on the MC80 transmembrane region and cytoplasmic tail. Tapasin degradation is accompanied by a loss of TAP, which limits MHC-I access to cytosolic peptides. Our findings reveal a unique mechanism by which MCV undermines adaptive immune surveillance.

## Author summary

The presentation of antigenic peptides by classical MHC-I to cytotoxic T-cells is a cornerstone of antiviral immunity. As such, viruses have devised a plethora of strategies to target MHC-I or cellular components involved in MHC-I antigen presentation in order to block effective T-cell surveillance. Molluscum contagiosum virus (MCV) is a human-specific poxvirus that produces skin lesions that can persist for months on a healthy individual. Herein, we demonstrate that MCV encodes a protein, MC80, which disrupts MHC-I antigen presentation by associating with the peptide loading complex, a set of proteins responsible for transporting peptides into the endoplasmic reticulum and loading them into a groove on MHC-I proteins. These MHC-I/peptide complexes are then trafficked to the plasma membrane for presentation to T-cell receptors. MC80 shares sequence similarity with classical MHC-I, and like MHC-I, requires β2m to function. We have found that MC80 engages a peptide loading complex specific chaperone, tapasin, which leads to its ubiquitination and subsequent degradation along with the TAP peptide transporter. This prevents peptides from being trafficked into the endoplasmic reticulum and severely limits peptide loading onto MHC-I. Thus, MCV-infected cells expressing MC80 can be protected from T cell killing due to the sabotage of cell surface MHC-I/peptide presentation.

## Introduction

Molluscum contagiosum virus (MCV) is a phylogenetically distinct poxvirus with a significant global disease burden [1,2]. MCV infections are thought to be restricted to humans, producing cutaneous lesions which often lack signs of an inflammatory response and persist for months to years in otherwise healthy individuals. This is in stark contrast to well characterized orthopoxviruses and parapoxviruses, which generally have a broader tropism and present as acute inflamed infections [1,3]. The persistence of MCV appears to be coupled to its ability to remain undetected by the immune system, as inflammatory responses have been implicated in spontaneous regression of MCV lesions [4,5]. Consistently, immunodeficient individuals are prone to exacerbated MCV infections [6,7]. The clinical features of MCV infections highlight a unique interplay between the virus and the human immune system.

MCV has clearly become well adapted to its niche, encoding a repertoire of proteins which are capable of evading the immune responses of the human epidermis. Yet, of the 59 open reading frames (ORFs) which distinguish MCV from orthopoxviruses [8,9], only nine have been well characterized [10,11,12,13,14,15,16,17,18]. Studies regarding the pathogenesis and immune evasion mechanisms employed by MCV have been severely limited by the lack of an animal model or cell line to propagate the virus [19]. Nevertheless, the ORFs unique to MCV likely play a major role in ensuring human-specific epidermal persistence, and should thus be more thoroughly characterized.

Among the MCV ORFs without a known function, MC80R shares moderate sequence similarity with the α1–3 domains of classical MHC-I. Given the central role of MHC-I and MHC-I-like proteins in inhibiting natural killer cells (NKs), it was long suspected that MC80 may be involved in NK subversion [9,20]. However, unlike classical MHC-I which is presented on the cell surface to NK and T cells, MC80 appeared to be retained in the endoplasmic reticulum (ER) [20]. As MC80 did not come to the cell surface, its host target and function have remained elusive.

Several large DNA viruses, particularly herpesviruses, have been found to repurpose the MHC-I fold in order to evade cell-mediated immune defenses. The specific function of each of these viral MHC-I-like proteins is highly related to its cellular localization. Cell surface viral MHC-I-like proteins (e.g. murine cytomegalovirus (MCMV) m157, human cytomegalovirus (HCMV) UL18) generally function as ligands for NK-inhibitory receptors without concurrently presenting viral peptides to cytotoxic T lymphocytes (CTL) [21]. However, intracellular MHC-I-like proteins (e.g. MCMV m145/m152/m155) are not directly exposed to NKs or CTLs. Instead, these proteins tend to localize to the ER/Golgi/lysosomal compartments where they retain or lead to the degradation of NK-activating ligands and, in some cases, classical MHC-I. Additionally, secreted viral MHC-I-like proteins have been identified which act as competitive antagonists of TNFα signaling and NKG2D-mediated NK activation (tanapox 2L and cowpox OMCP, respectively) [22,23,24,25]. Thus, through prolonged co-evolution, large DNA viruses have used the MHC-I fold for a diverse array of immune evasion functions.

As opposed to viral MHC-I-like proteins, vertebrates utilize classical MHC-I to display a repertoire of peptides on the cell surface to surveilling CTLs [26,27]. This pivotal role demands rapid yet stringent quality control in the assembly of MHC-I/peptide complexes. To accomplish this, host cells utilize a multi-subunit peptide loading complex (PLC); comprised of tapasin (Tpn), transporter associated with antigen processing (TAP), ERp57, and calreticulin (CRT) [28,29]. In the ER, nascent MHC-I heavy chains (HC) are initially stabilized by a chaperone, calnexin (CNX), and subsequently by heterodimerization with β2m [30]. However, HC/β2m must assemble with a high-affinity peptide, usually via the PLC, in order to efficiently traffic from the ER to the cell surface [31]. HC/β2m accomplishes this by directly binding Tpn/ERp57, which plays a central role in both transiently stabilizing the unloaded conformation of MHC-I and bridging the interaction between MHC-I and TAP [32,33]. TAP functions by transporting short cytosolic peptides into the ER, allowing PLC-associated MHC-I to sample the repertoire of proteasome-degraded proteins within the cell [34,35]. Once an MHC-I molecule has been loaded with a high-affinity peptide, it dissociates from the PLC and traffics to the cell surface. Given the critical role played by the PLC in peptide loading, both MHC-I itself and components of the PLC provide attractive targets for viruses seeking to subvert CTL responses [28]. However, the magnitude of MHC-I downregulation depends on the specific target and the MHC-I alleles expressed by the host cell. Understanding these viral mechanisms provides insight into the pathogenesis of viral infections, as well as the underlying cellular pathways that these viruses exploit.

Here we demonstrate that expression of MC80 results in ER-retention and consequent surface downregulation of classical MHC-I in human and mouse cells. Mechanistically, we found that MC80 interacts with Tpn via its luminal domain and targets Tpn for ER-associated degradation in a transmembrane (TM)- and cytoplasmic tail-dependent manner. The loss of Tpn coincides with a loss of TAP, further impeding the assembly of MHC-I with high-affinity peptides. Our findings reveal a strategy employed by MCV to disrupt antigen presentation and thereby CTL responses by exploiting MHC-I fold recognition by the PLC.

## Results

### MCV-encoded MC80 downregulates surface MHC-I

**Fig 1:**
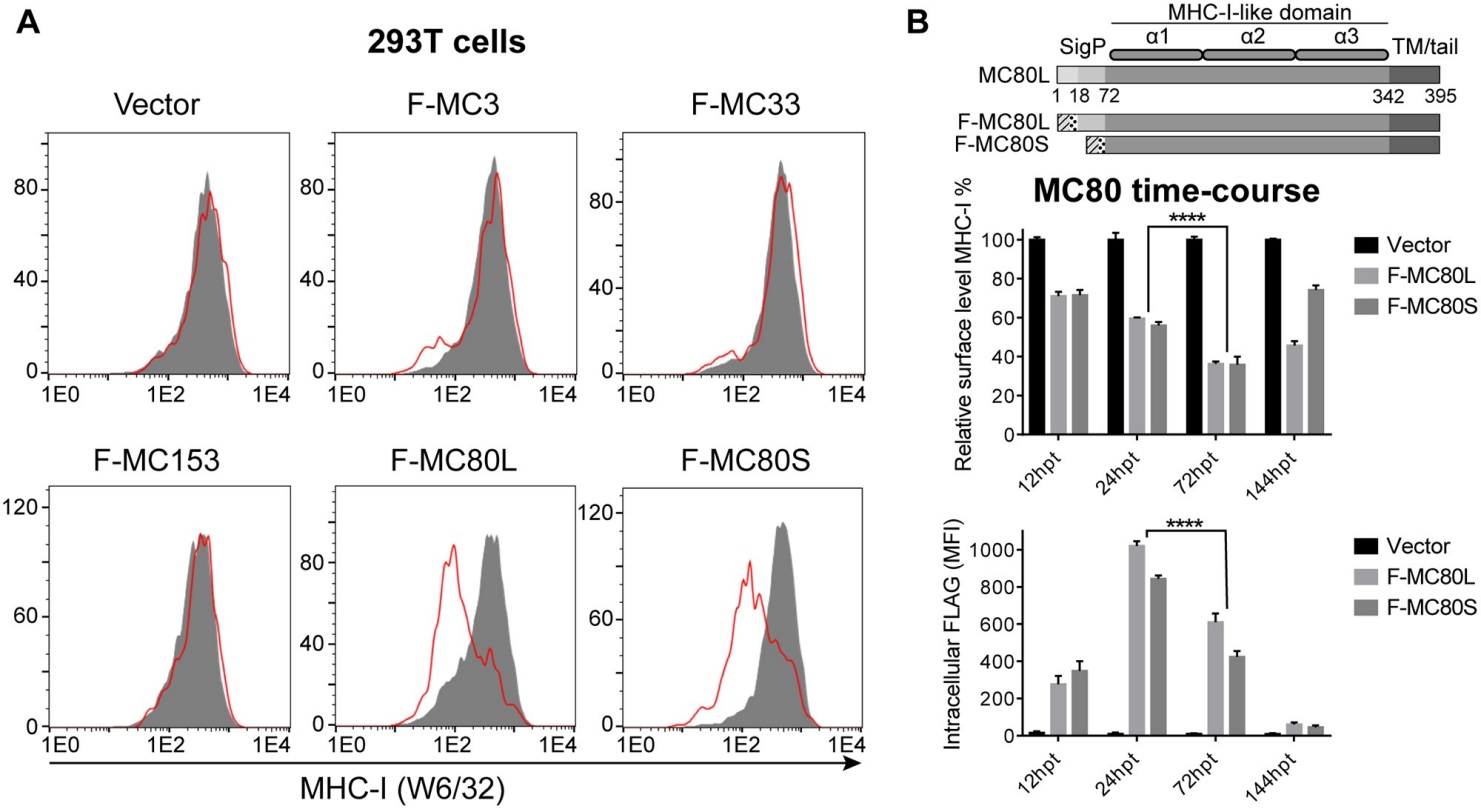
MC80 downregulates MHC-I surface expression. (A) The four MCV-specific ORFs which are predicted to encode a type-1 transmembrane protein were cloned into the pMXsIG vector and transiently transfected into HEK 293T cells 2–4 days prior to analysis of MHC-I (W6/32) surface expression by flow cytometry (three independent experiments of two replicates each). (B) The top panel is a schematic of MC80 constructs used in A and B, where diagonal lines indicate the signal peptide derived from mouse β2m, followed by dotted regions indicating N-terminal Flag peptide, and finally the MC80 ORF 18–395 (F-MC80S) or 72–395 (F-MC80L), as described in the Materials and Methods. HEK 293T cells were transiently transfected with a vector control or Flag-tagged MC80 constructs. Cells were stained for MHC-I surface expression (middle panel) and intracellular Flag expression (bottom panel) 12 hours post-transfection (hpt), 24hpt, 72hpt, and 144hpt. Error bars represent the standard deviation from three independent replicates. Statistical analyses were performed on the effect of each MC80 construct individually, with equivalent significance found for both.

While MCV does not encode an ORF with sequence-similarity to any viral protein known to downregulate MHC-I, previous studies have suggested that MCV may be downregulating MHC-I and β2m in human lesions [5,36]. Given that some poxviruses do not appear to subvert MHC-I antigen presentation [1,37], we hypothesized that the MCV ORF(s) responsible for MHC-I downregulation may be unique to MCV. Additionally, as MHC-I traffics through the ER/Golgi to the plasma membrane, we limited our initial screen to the four MCV-specific ORFs which are predicted to encode type-1 transmembrane proteins (MC3, MC33, MC80, and MC157). We cloned the respective MCV-1 variants into an IRES-GFP retroviral vector (pMXsIG), replacing each predicted signal peptide with the mouse β2m signal peptide and an N-terminal Flag tag. Following transient transfection of human embryonic kidney (HEK-293T) cells with these constructs or vector control, we found that MC80 dramatically decreased the level of cell surface MHC-I by 2–4 days post-transfection (Fig 1A).

MC80 is well conserved among known MCV strains, sharing 24–36% amino acid identity to the ectodomains of human classical and non-classical MHC-I. The MC80 ORF has at least two potential start codons N-terminal to the MHC-I like α1 domain, termed MC80L and MC80S, which both provide unusually long signal peptides (S1A Fig). While the ectodomains of MC80 share moderate sequence-similarity with MHC-I, functionally distinct regions exhibit varying levels of conservation (S1A Fig). Briefly, residues involved in peptide binding and PLC interactions are not well conserved between MC80 and classical MHC-I (S1A Fig) [38,39,40]. In contrast, residues known to be involved in β2m binding are well-conserved, consistent with a previous study which found that MC80 associates with β2m [20,41]. This same study found that MC80 did not traffic to the cell surface, which we were able to recapitulate by flow cytometry and EndoH sensitivity assays, indicating that MC80 is retained in the ER (S1B and S1C Fig).

The cell surface half-life of MHC-I can be greater than 24 hours depending on the specific cell line and peptide(s) displayed [42,43]. We investigated the kinetics of MHC-I surface downregulation using a transient transfection system, finding that maximal expression of MC80 at 24 hours post transfection (hpt) did not coincide with maximal downregulation of MHC-I surface expression (Fig 1B). Instead, we found a significantly lower level of surface MHC-I at 72hpt than 24hpt, even though there was less MC80 at the later time point. Additionally, our data demonstrated that continued expression of MC80 was necessary to maintain MHC-I downregulation (Fig 1B). As extended HLA class I half-lives may have played a role in the apparent time-dependence of MC80-mediated MHC-I downregulation (Fig 1B), we employed a stable retroviral transduction system to achieve a steady state of MHC-I downregulation in further analyses. This time-dependence may also provide insight into why the previous MC80 study reported no change in surface expression of HLA-A2 12 hours post-infection of an MC80-expressing vaccinia virus [20].

### MC80 specifically downregulates surface expression of peptide-binding MHC-I

**Fig 2:**
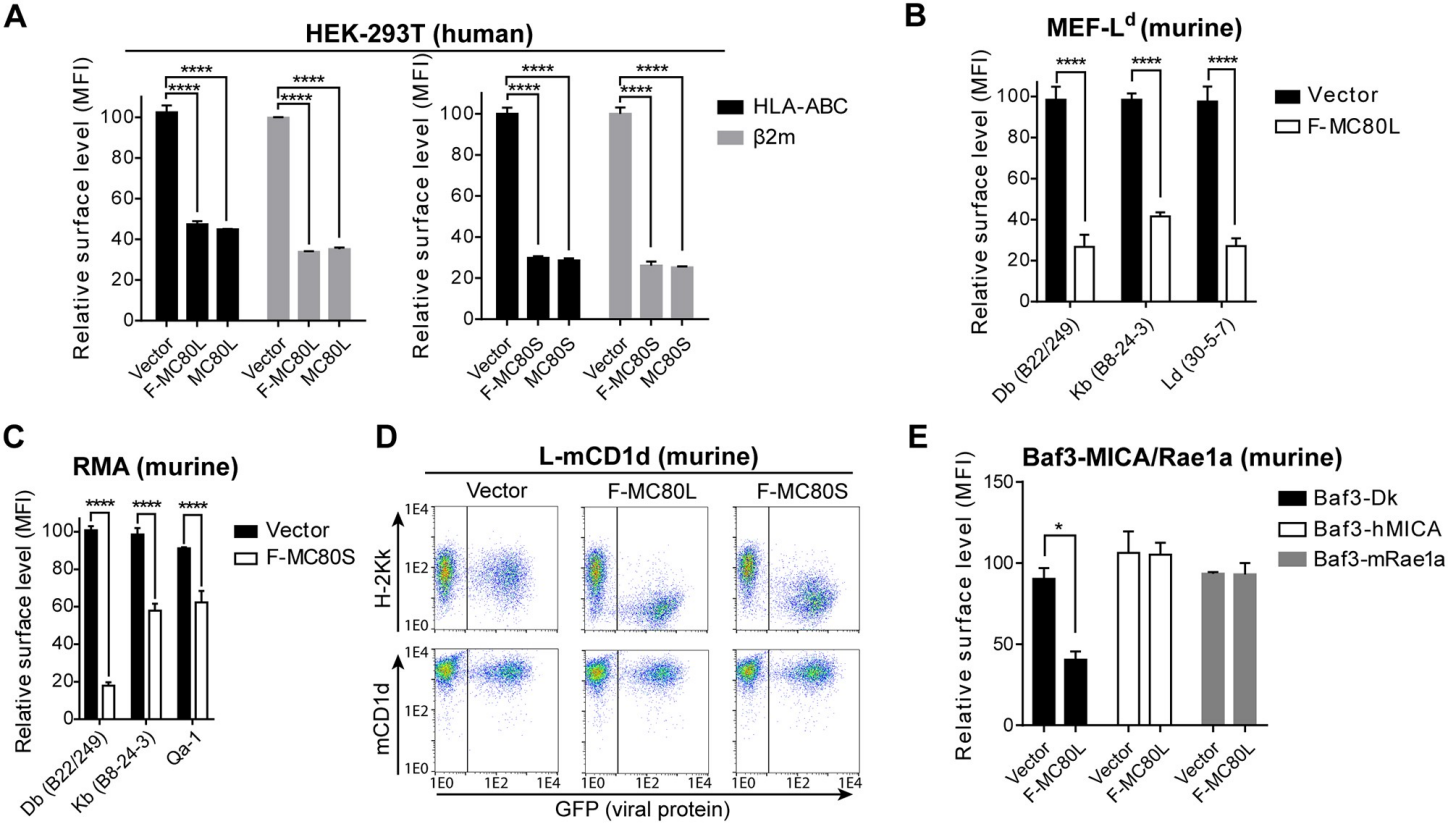
MC80 specifically downregulates peptide-binding MHC-I in human and murine cell lines. (A) The downregulation of MHC-I and β2m (examined using either anti-HLA-ABC (W6/32) or anti-β2m) by F-MC80L and F-MC80S was compared to respective untagged MC80 constructs in transiently transfected HEK 293T cells, two independent replicates each. (B) Using C57BL/6-derived MEFs stably expressing L^d^, the specific surface expression of D^b^, K^b^, and L^d^ MHC-I alleles were determined in the presence of F-MC80L or vector-control, two independent replicates each. (C) Using RMA cells, the surface expression of D^b^, K^b^, and Qa-1 were determined in the presence of retrovirally transduced F-MC80S or vector-control, two independent replicates each. (D) Using L cells stably expressing mouse CD1d, the surface expression of K^k^ and CD1d were determined in the presence of retrovirally transduced F-MC80S/L, two independent replicates each. (E) Using murine Baf3 cells stably expressing human MICA or murine Rae1α, the surface expression of D^k^, hMICA, and mRae1α were determined in the presence of retrovirally transduced MC80L or vector-control. Error bars represent the standard deviation from two independent replicates.

Since viral MHC-I evasion mechanisms can downregulate MHC-I with varying levels of promiscuity [44,45], we next sought to determine the specificity of MC80-mediated MHC-I downregulation. Flow cytometry analysis demonstrated that expression of MC80 markedly decreased the surface levels of classical MHC-I in multiple human cell lines, including HEK 293T, Hela (human cervical cancer cell line), and HFF-1 (a human foreskin fibroblast cell line) (Fig 2, S2 Fig). A comparable effect was observed with untagged MC80 constructs with the canonical signal peptide, indicating that the Flag tag did not significantly affect MC80 function (Fig 2A). Interestingly, MC80 also downregulated all tested alleles of classical MHC-I in murine cell lines (Fig 2B, 2C, 2D and 2E). Additionally, of the non-classical MHC-I proteins examined, MC80 significantly decreased the surface expression of Qa-1 but did not significantly affect surface expression of CD1d or the NKG2D ligands MICA and Rae1a (Fig 2C, 2D and 2E). Like classical MHC-I and Qa-1, CD1d requires β2m for stable expression, indicating that MC80 is unlikely to function by competing for β2m. Instead, MC80 appears to be specifically downregulating peptide-binding MHC-I through a cellular component/pathway that is conserved between humans and mice.

### MC80 impairs PLC-mediated peptide loading

**Fig 3:**
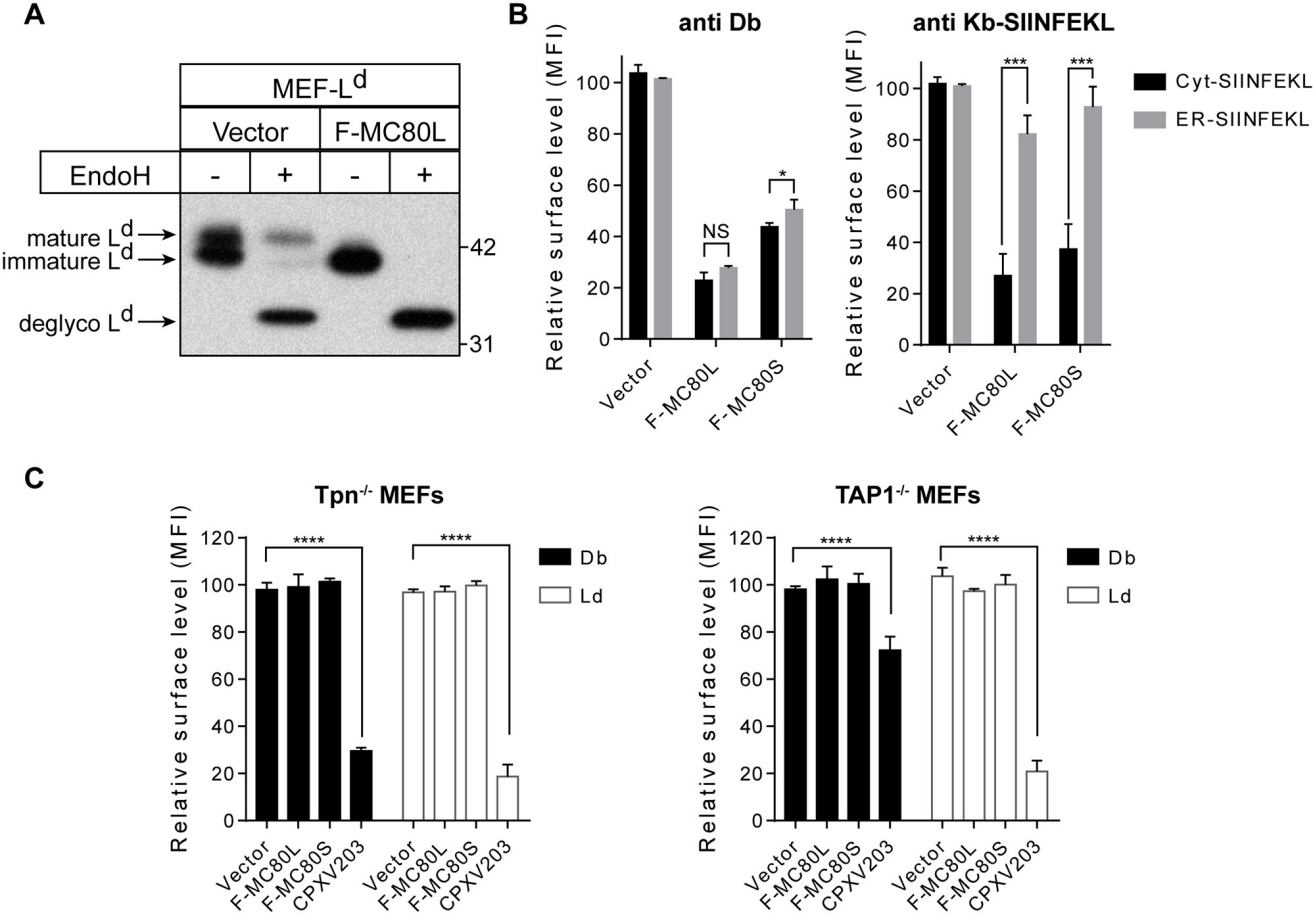
MC80 prevents the maturation of MHC-I through dysregulation of peptide loading. (A) MEF-L^d^ cells expressing vector control or F-MC80L were immunoprecipitated with anti-L^d^ followed by an EndoH-sensitivity assay, with equivalent results demonstrated in two independent replicates. Ladder markers for western blots indicate the protein mass in kilodaltons. (B) MEF cells stably expressing Cyt-SIINFEKL or ER-SIINFEKL were transduced with a vector control, MC80L, or MC80S and stained for D^b^, or K^b^-SIINFEKL. Error bars represent the standard deviation from two independent replicates. (C) Tpn^-/-^ or TAP^-/-^ MEFs expressing vector control, MC80L-F, MC80S-F, or CPXV203 were stained for D^b^ and L^d^. Quantification of Tpn^-/-^ and TAP^-/-^ MEF surface MHC-I is relative to the respective vector controls, which is considerably lower than MHC-I surface expression on wild-type MEFs. All constructs were stably transduced using retroviral systems. Error bars represent the standard deviation from two independent replicates.

As viruses are well known to have strategies to downregulate classical MHC-I by altering the cellular trafficking of MHC-I [28], we next examined the maturation state of L^d^ by EndoH sensitivity in the presence or absence of MC80 (Fig 3A). We found that MC80 did not appear to affect the steady state expression level of L^d^. However, compared to the 35% of EndoH-resistant L^d^ in the control, we found that L^d^ was completely EndoH-sensitive in MC80-expressing cells (Fig 3A, S3A Fig). This indicates that MC80 interferes with MHC-I trafficking to the Golgi, which consequently decreases the extent of surface MHC-I.

Virally-encoded MHC-I saboteurs are further dichotomized into PLC-dependent and PLC-independent mechanisms; as exemplified by the cowpox virus CPXV012 and CPXV203 proteins, respectively [46,47,48,49]. To determine which strategy is utilized by MC80, we next tested whether MC80 downregulates MHC-I in murine embryonic fibroblasts (MEF) expressing SIINFEKL, a K^b^-specific peptide of egg ovalbumin, either in the cytosol or in the ER. Cytosolic SIINFEKL requires TAP to be transported into the ER for MHC-I loading, whereas ER-SIINFEKL is able to load onto MHC-I independent of TAP function. Because CPXV203 does not require the PLC in order to downregulate MHC-I surface expression, it dramatically affects K^b^/SIINFEKL expression in both cell lines (S3B Fig). However, CPXV012 only induces significant downregulation of K^b^/SIINFEKL in cells expressing cytosolic SIINFEKL, as its mechanism of action is dependent on TAP [49]. Similar to CPXV012, MC80-mediated downregulation of K^b^/SIINFEKL could be rescued by expression of SIINFEKL in the ER (Fig 3B, S3B Fig). SIINFEKL localization had only a marginal effect on the surface expression of D^b^ in the presence of MC80, indicating that this effect was specific to K^b^/SIINFEKL (Fig 3B).

While TAP- and Tpn-deficient cells display low levels of MHC-I on the cell surface, these levels can be further decreased by PLC-independent viral mechanisms, such as CPXV203. However, we found that MC80 functionally relies on the presence of TAP and Tpn, as murine MHC-I alleles were not further downregulated by MC80 in TAP- or Tpn-deficient cells, relative to vector control (Fig 3C). Together, (1) the lack of MHC-I-maturation, (2) the specific rescue of MHC-I by an ER targeted peptide, and (3) the TAP-/Tpn-dependence collectively suggest that MC80 sabotages the PLC-assisted peptide transport/loading of MHC-I in the ER.

### The luminal domain of MC80 is sufficient for PLC-association but not MHC-I downregulation

**Fig 4:**
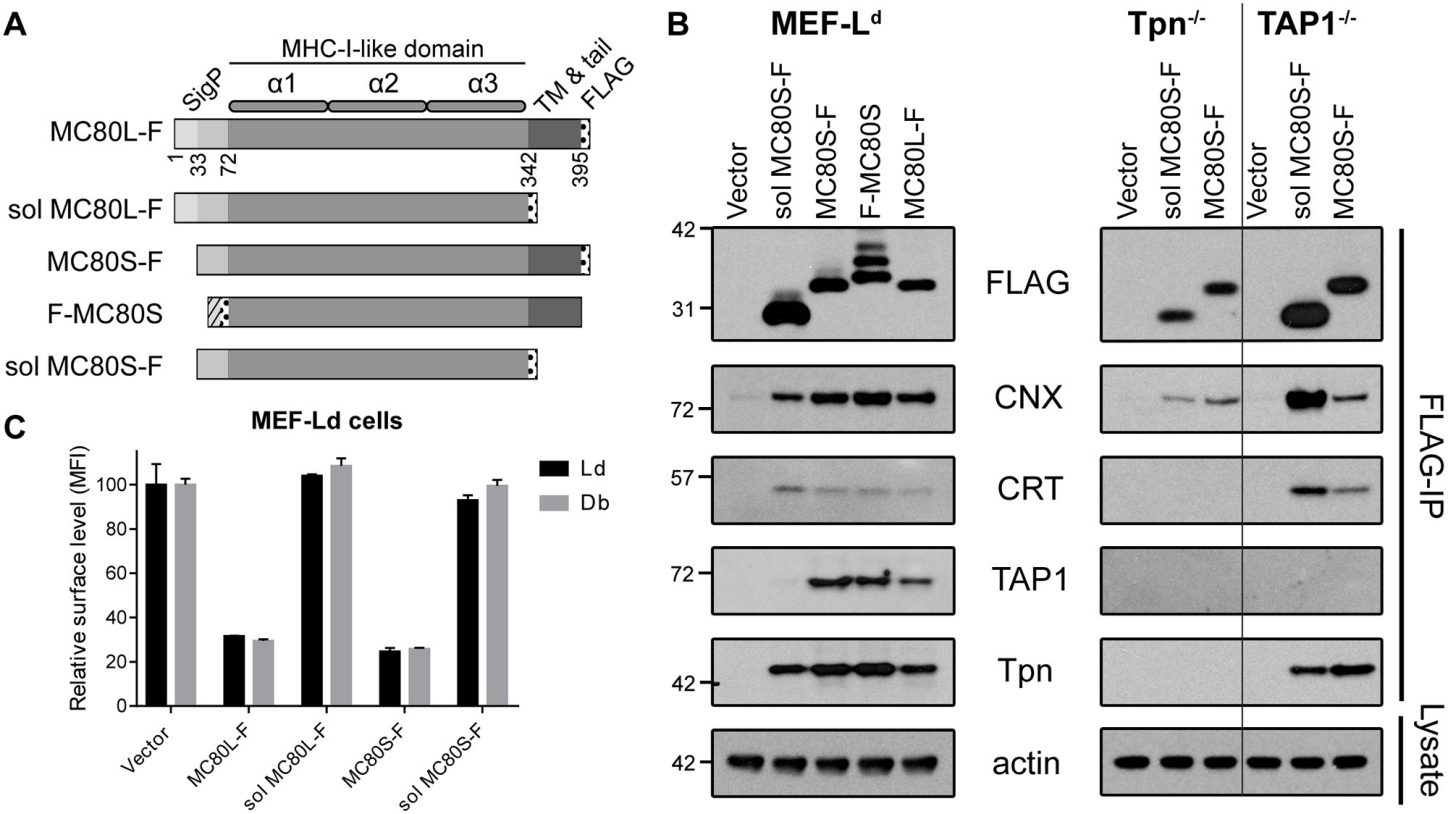
MC80’s luminal domain associates with the PLC primarily through Tpn, but the transmembrane and/or tail is necessary for MHC-I downregulation. (A) Schematic depiction of MC80 constructs fused with N-terminal or C-terminal Flag-tags, truncating the extended signal peptide and/or the transmembrane and tail as described in the Materials and Methods. The dashed box indicates mouse β2m signal peptide while dotted boxes indicate the location of the Flag peptide. (B) MEF-L^d^ (untreated), Tpn^-/-^ (+IFNɣ), and TAP^-/-^ (+IFNɣ) cells retrovirally transduced with the indicated MC80 constructs or vector control were lysed and immunoprecipitated with anti-Flag antibody. Flag-IP eluants were immunoblotted for Flag, CNX, CRT, TAP1, and Tpn. Data are representative of two independent replicates. Ladder markers for western blots indicate the protein mass in kilodaltons. (C) MEF-L^d^ cells expressing the indicated MC80 constructs or vector control were stained for L^d^ and D^b^, and quantified relative to vector control by flow cytometry, representative of two independent replicates, with two technical replicates each.

Given the central role of Tpn in peptide loading and the partial conservation of Tpn-binding residues within MC80 (S1A Fig), we hypothesized that MC80 may subvert peptide loading by competitively binding Tpn to block the interaction between MHC-I and the PLC. A Flag-IP of MC80 followed by western blotting (WB) for PLC components supported this hypothesis; demonstrating that Tpn, TAP, CRT and CNX co-immunoprecipitate (co-IP) with the full length MC80 constructs (Fig 4B). While the N-terminal Flag-tag did not appear to impact MHC-I downregulation in Fig 2A, we observed that an anti-Flag WB of F-MC80S (N-terminally Flag-tagged MC80S) produced a laddering effect in non-reduced samples. Therefore, we used C-terminal Flag-tagged constructs, encoding the canonical signal peptide of MC80, for all further co-IP experiments.

Notably, a truncated MC80S protein which lacks the putative TM and cytoplasmic tail (sol MC80S-F) could also co-IP Tpn, CRT and CNX but not TAP1 in murine cells (Fig 4B). This suggests that MC80 primarily associates with the PLC via the luminal domain and that the interaction between MC80 and TAP1 may be further stabilized by the TM and cytoplasmic tail. Using TAP1-deficient MEFs treated with interferon gamma (IFNɣ), we were able to recapitulate the association of the MC80 luminal domain with Tpn, CRT and CNX. However, in the absence of Tpn, we could not detectably co-IP TAP1 or CRT with MC80, even when the Tpn^-/-^ MEFs were treated with IFNɣ. These data suggest that MC80 interacts with CNX and Tpn via the luminal domain; and the latter association may bridge the interaction of MC80 with CRT and TAP1, reminiscent of classical MHC-I assembly in the ER. A co-IP of PLC components with MC80 in 293T cells demonstrated that soluble MC80S-F interacts with both Tpn and TAP1, further suggesting that the TM and tail of MC80 may not be necessary for the association of MC80 and TAP (S4A Fig). Despite the observed PLC-associations, flow cytometry demonstrated that soluble MC80 constructs do not markedly downregulate surface MHC-I in MEFs or HFF-1s (Fig 4C, S2B Fig). As binding to the PLC appears to be insufficient for MC80 function, Tpn-competition/blockade is unlikely to be the mechanism of MHC-I downregulation, as we had hypothesized.

### The destabilization of Tpn and TAP requires the MC80 TM and cytoplasmic tail as well as β2m

**Fig 5:**
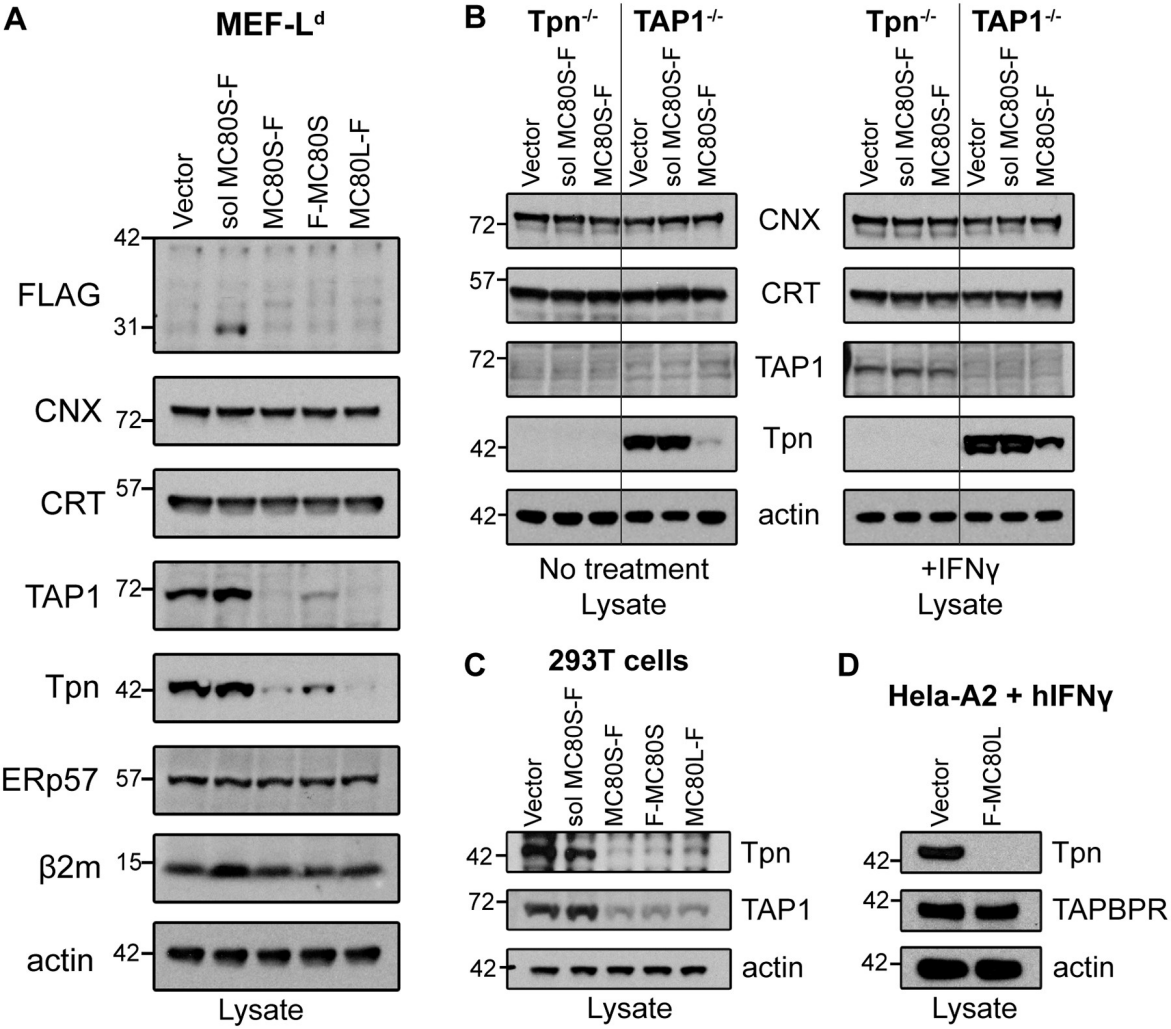
MC80 induces the degradation of Tpn independent of TAP, and induces the degradation of TAP in a Tpn-dependent manner. (A) MEFs were retrovirally transduced with the indicated MC80 constructs or vector-control were lysed and the lysates were immunoblotted for Flag, CNX, CRT, TAP1, Tpn, ERp57, β_2_m and actin, representative of at least two replicates each. (B) Tpn^-/-^ and TAP1^-/-^ MEFs were retrovirally transduced with the indicated MC80 constructs or vector-control, and either incubated with or without mIFNɣ for 24hr. The Tpn/TAP1-deficient MEFs were then lysed, and lysates were immunoblotted for CNX, CRT, TAP1, Tpn, and actin. Untreated Tpn/TAP1-deficient MEFs are representative of two independent replicates, and the IFNɣ-treated Tpn/TAP1-deficient MEFs are representative of three independent replicates. (C) HEK 293T and (D) Hela-A2 cells were retrovirally transduced with MC80 constructs or vector control. (C) Tpn and TAP1 were found to be downregulated by MC80 in HEK 293T cells. Additionally, soluble MC80 does not downregulate either Tpn or TAP1. Blots are representative of two independent replicates. (D) Hela-A2 cells were treated with 100U/mL hIFNɣ for 24-48hr followed by immunoblotting the lysate for the indicated proteins. Tpn was undetectable in the presence of F-MC80L, while TAPBPR remained unchanged by F-MC80L. Blots are representative of at least two independent experiments. Ladder markers for western blots indicate the protein mass in kilodaltons.

To determine the role of the transmembrane and tail in MC80-mediated MHC-I downregulation, we assessed the steady state levels of PLC components in the presence of various MC80 constructs. Remarkably, we found that MC80L and MC80S, but not soluble MC80S, dramatically reduced the steady state levels of Tpn and TAP compared with vector control in MEFs and HEK 293Ts (Fig 5A and 5C). However, CNX, CRT, and ERp57 were not downregulated by the expression of MC80 in MEFs. While the soluble MC80S construct slightly increased β_2_m levels, they appeared unchanged by active forms of MC80. These data suggest that MC80 selectively destabilizes Tpn and TAP in a TM/tail-dependent manner. While full-length forms of MC80 downregulated Tpn, the completion of tapasin degradation appears to be cell-line specific. For instance, the MEF-L^d^ cells in Fig 4B (left panel) were not treated with drug or cytokine to rescue Tpn levels, yet all MC80 constructs associated with Tpn. However, only soluble MC80S-F appeared to associate with Tpn in untreated TAP^-/-^ MEFs and 293T cells, presumably due to the degradation of Tpn by the functional MC80 constructs (S4 Fig). Therefore, to demonstrate the ability of functional MC80 to associate with Tpn in the absence of TAP, we treated the Tpn^-/-^ and TAP^-/-^ MEFs with mIFNɣ (Fig 4B; right panel). F-MC80L also downregulated Tpn in Hela cells in the presence of IFNɣ, but did not markedly affect the steady-state levels of TAPBPR, a structural relative of Tpn known to interact with classical MHC-I (Fig 5D) [40].

Given the interdependence of Tpn and TAP, we next sought to determine whether MC80 primarily targeted one component or both equivalently. Using TAP1^-/-^ MEFs, we were able to recapitulate the MC80 TM/tail-dependent degradation of Tpn observed in wildtype MEFs (Fig 5B). However, the level of TAP in Tpn^-/-^ cells expressing MC80 was comparable to the vector control. This was more readily observable when the Tpn^-/-^ cells were treated with IFNɣ for 24hr prior to harvesting, to upregulate TAP expression (Fig 5B, right panel). Thus, while the destabilization of TAP by MC80 depends on the presence of tapasin, the MC80-mediated loss of tapasin is independent of TAP, indicating that tapasin is the primary target of MC80 in murine cells. TAP destabilization is potentially a consequence of the loss of Tpn, given that both this and previous studies demonstrate that TAP is generally unstable in the absence of Tpn (Fig 5B, left panel) [50,51]. We hypothesize that tapasin is also the primary target in human cells due to the homology of murine and human PLC components. However, as murine TAP is apparently more Tpn-dependent than human TAP, our data does not rule out the possibility that MC80 directly targets TAP for degradation in human cells.

**Fig 6:**
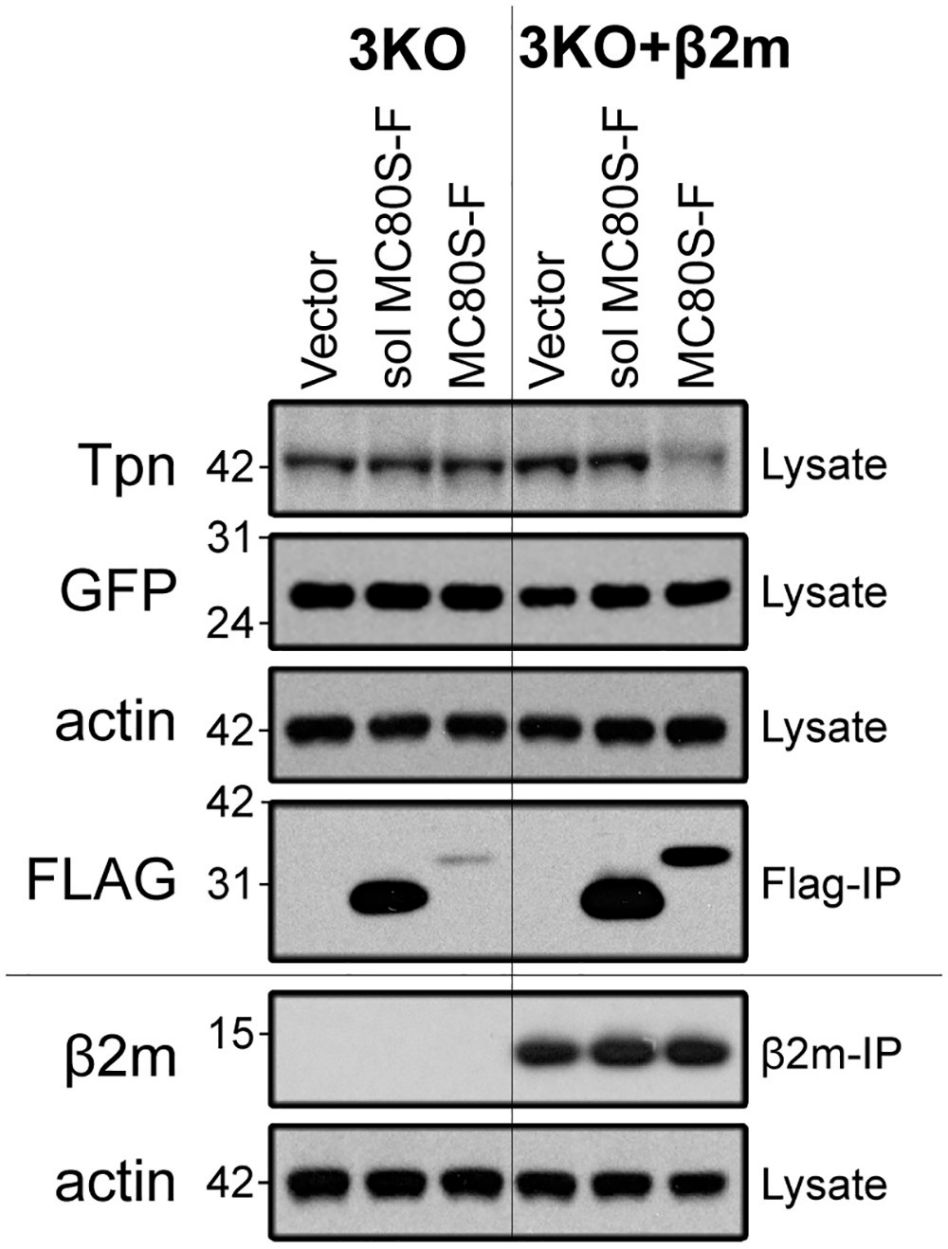
β2m is required for the MC80-mediated degradation of Tpn. MEF cells derived from D^b^/K^b^/β2m triple knock-out (3KO) mice were trans-complemented with β2m (3KO+β2m). Both the 3KO and 3KO+β2m cells were retrovirally transduced with vector control, soluble MC80S-F, or MC80S-F. Lysates were either directly western blotted for Tpn, GFP, and actin, or immunoprecipitated with anti-Flag or anti-β2m, followed by western blotting for the respective immunoprecipitant. Three independent replicates were performed (2 FLAG-IP, 1 β2m-IP). Ladder markers for western blots indicate the protein mass in kilodaltons.

While previous work demonstrated that MC80 associates with β2m, it was not clear whether this association was functionally relevant. To determine whether β2m was necessary for MC80-mediated downregulation of Tpn, we used a classical MHC-I (H2-K^b^/H2-D^b^) and β2m triple knock-out MEF cell line (3KO) with or without stably transduced β2m. As observed in wild-type MEFs, the soluble form of MC80S-F did not cause Tpn degradation in either cell line. However, β2m expression was necessary for MC80S-F to induce Tpn degradation (Fig 6). In addition, the level of MC80 in 3KO cells without β2m is lower than that in 3KO cells with β2m transcomplementation. Given that the cistronically-translated GFP was expressed at similar levels, these findings indicate that, (1) β2m can stabilize MC80, (2) β2m is required for MC80 function, and (3) MC80-mediated degradation of Tpn is classical MHC-I-independent.

### MC80-mediated Tpn degradation is proteasome dependent

**Fig 7:**
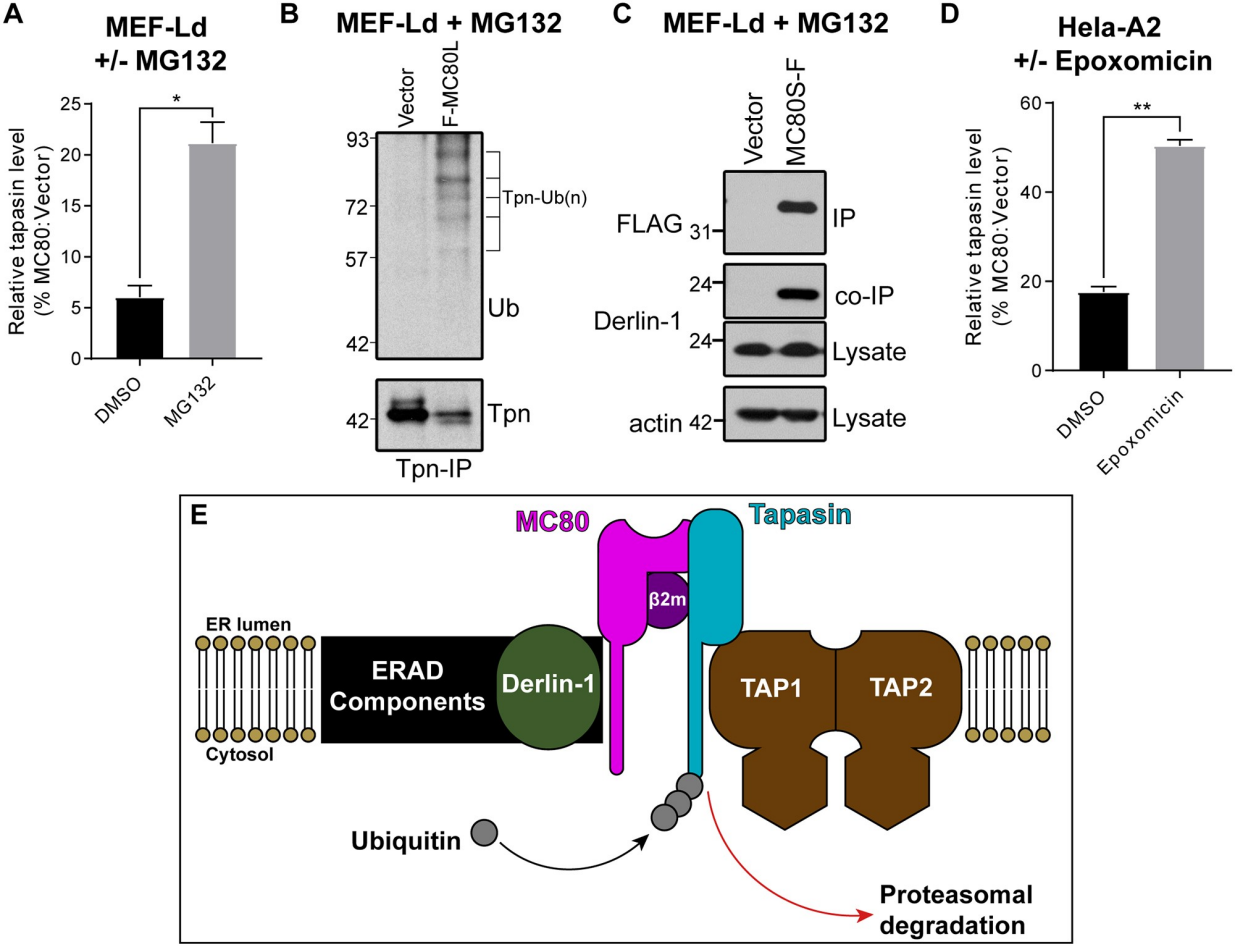
MC80 induces the degradation of Tpn through the ubiquitin-proteasomal pathway. (A) MEF-L^d^ cells stably expressing vector control or MC80L-F were treated with mIFNɣ for 24hr and with MG132 or DMSO control for 9hr before being subjected to WB for Tpn. Each Tpn band from MC80-expressing cells was quantified as a percentage of the respective vector control Tpn band. Error bars represent the standard deviation from two independent replicates. (B) The lysates of MEF-L^d^ cells expressing vector control or F-MC80L were treated with mIFNɣ and MG132 as in (A) and immunoprecipitated with anti-Tpn antibody, followed by Tpn- and ubiquitin-WBs, representative of four independent replicates. Ladder markers for western blots indicate the protein mass in kilodaltons. (C) MEF-L^d^ cells stably expressing vector control or MC80S-F were treated with MG132 for 8 hours, followed by FLAG-IP. The elutants were immunoblotted for FLAG and Derlin-1, while the lysates were immunoblotted for Derlin-1 and actin. Blots are representative of two replicates. (D) Hela-A2 cells stably expressing vector control or MC80S-F were treated with hIFNɣ for 40 hr followed by Epoxomicin or DMSO control for 9hr before being subjected to WB for Tpn. Each Tpn band from MC80-expressing cells was quantified as a percentage of the respective vector control Tpn band. Error bars represent the standard deviation from two independent replicates. (E) Proposed mechanism for MC80-mediated subversion of MHC-I presentation. The cartoon depicts MC80 interacting with the PLC components Tpn and TAP in a manner similar to classical MHC-I, but also recruiting host ERAD machinery to trigger the ubiquitination and subsequent degradation of tapasin.

The majority of eukaryotic protein degradation is mediated through proteasomal and lysosomal pathways [52]. Autophagy has also been implicated in trafficking proteins from the ER to the lysosome/autophagosome for degradation [53]. To determine which host degradation pathway was being exploited by MC80, we assessed the effects of two inhibitors of proteasomal degradation (MG132 and Epoxomicin) and one inhibitor of lysosomal degradation (chloroquine). We also assessed an Atg5^-/-^ murine microglial cell line, which is deficient in classical autophagy. Due to the toxicity of the tested drugs and the slow intrinsic turnover of Tpn, we treated the cells with IFNɣ prior to drug exposure to increase the synthesis of Tpn. Following a nine hour incubation, MG132 treatment partially but significantly rescued the expression of Tpn in the presence of MC80 in murine cells (Fig 7A, S5A Fig). The proteasome-dependence of MC80 was also demonstrated in hIFNɣ-stimulated Hela (human) cells using the more specific inhibitor, Epoxomicin (Fig 7D, S5C Fig). In contrast, neither the Atg5^-/-^ cell line nor chloroquine treatment had a discernable effect on MC80 function (S5A and S5B Fig). Furthermore, upon treatment with IFNɣ and MG132, anti-Tpn antibody co-immunoprecipitated ubiquitinated bands corresponding to the size of multi/poly-ubiqutinated Tpn, specifically in the presence of MC80 (Fig 7B). This suggests that the expression of MC80 leads to the ubiquitination of Tpn. Aside from ubiquitination, ER-associated degradation (ERAD) requires retrotranslocation of targeted proteins to the cytoplasm for proteasomal degradation to occur. While multiple retrotranslocation complexes exist in the ER, we have observed that MC80 associates with Derlin-1 in the presence of MG132 (Fig 7C). Given that MC80 is retained in the ER, these data suggest that MC80 selectively destabilizes Tpn by recruiting ER-associated degradation (ERAD) components for the ubiquitination and retrotranslocation of Tpn (Fig 7E).

## Discussion

Through this study, we demonstrate that (1) the MHC-I-like MCV protein, MC80, associates with the PLC via its luminal domain; (2) MC80 remains localized to the ER, where it induces the degradation of Tpn and TAP to impede peptide loading and consequent MHC-I surface expression; (3) MC80 requires β2m to degrade Tpn; (4) MC80 primarily targets Tpn, with our data suggesting that MC80 induces ERAD through a mechanism that requires its transmembrane and cytoplasmic tail. Taken together, these findings support a model wherein MC80 directly interacts with Tpn via its luminal domain and presumably recruits cellular ERAD machinery via its transmembrane and/or tail to facilitate the degradation of Tpn; which secondarily destabilizes TAP. The loss of Tpn/TAP in turn dramatically affects the ability of nascent MHC-I to load high affinity peptides and subsequently traffic to the cell surface. Our experiments indicate that MC80L and MC80S both downregulate classical MHC-I, associate with Tpn, and degrade Tpn/TAP in human and mouse cells. Thus, the extended MC80 signal peptide does not appear necessary for MHC-I sabotage but may have an as-yet-unknown independent function. To our knowledge, this is the first example of a viral protein that primarily targets Tpn for degradation.

Given the central role of Tpn in PLC organization and function, it is not surprising that multiple virally-encoded proteins have been found to undermine its function to evade CTL killing. HCMV US3 directly competes for Tpn binding to prevent peptide loading, while adenovirus E3-19K obstructs the TAP interface to prevent Tpn from bridging classical MHC-I to the PLC [54,55]. Unlike these mechanisms, our data indicates that soluble MC80 can associate with the PLC, but does not prevent MHC-I presentation. Therefore, at the expression levels tested in our retroviral system, MC80 does not appear to be functioning as a competitive inhibitor of PLC-mediated peptide-loading. Instead, we find that Tpn is degraded in the presence of MC80; but Tpn levels can be partially rescued in MC80-expressing cells by inhibiting proteasomal degradation. We also found that Tpn was multi/poly-ubiquitinated in the presence of MC80, suggesting that MC80 induces the ER-associated degradation of Tpn. The fact that we can detect the association between functional MC80 and Tpn in our wild-type MEFs and interferon-induced TAP^-/-^ cells indicates that the downstream steps of ERAD (ubiquitination, retrotranslocation, and/or degradation) may be rate-limiting. However, this hindrance may be cell-line/species specific, as the association between MC80 and tapasin is only detectable for soluble (non-functional) MC80 in HEK 293T cells and untreated TAP^-/-^ MEFs. In comparison to other viral MHC-I-evasion mechanisms which utilize ERAD, HCMV US2/US11 are only known to target MHC while MHV68 mK3 primarily targets MHC-I with only a slight effect on TAP levels [56,57]. Recently, a virally encoded ER-resident ubiquitin E3 ligase, RHVP pK3, was found to degrade MHC-I, Tpn, and TAP [58]. However, the degradation of Tpn and TAP were found to be secondary effects of the pK3-mediated degradation of MHC-I. Conversely, MC80 appears to degrade Tpn independent of TAP or MHC-I. Thus, the MC80-mediated destabilization of Tpn is distinct from other known viral MHC-I-evasion mechanisms.

While US2 and US11 specifically target MHC, their mechanisms are reminiscent of MC80. Indeed, while none of these viral proteins appear to have a ubiquitin E3 ligase domain, all three appear to induce ubiquitination-mediated ERAD. The luminal domains of US2 and US11 are able to associate with MHC-I, but without their transmembrane or tail domains these viral proteins cannot induce MHC degradation [59,60]. The transmembrane and tail sequences that distinguish US2 and US11 are thought to be responsible for recruiting distinct ERAD pathways. Of note, Derlin-1 associates with US11 and is essential for US11-mediated, but not US2-mediated, ER-associated degradation of MHC-I [61,62,63]. Intriguingly, MC80 contains two glutamic acid residues in its predicted TM domain [64], which are exceedingly rare in human type I TM proteins [65]. While MC80 is also found to associate with Derlin-1, the observation that neither US2 nor US11 TMs contain a negatively-charged residue raises the question of whether MC80 co-opts a distinct ERAD pathway. The current data cannot rule out the possibility that MC80 is able to actively degrade TAP through the proximal interaction with Tpn or direct interaction with TAP. However, considering that the loss of Tpn has been previously shown to destabilize TAP in human and murine cells, it is attractive to speculate that the loss of TAP is a secondary effect of specific ubiquitination and degradation of Tpn [50,51].

Interestingly, MC80 is retained in the ER despite lacking a putative ER-retention motif [66]; even when expressed as a truncated protein without a TM or tail. MC80's ability to associate with β2m and members of the PLC suggest that it maintains an MHC-I-like fold, and thus may exploit host machinery which canonically retains unloaded MHC-I in the ER. The association of soluble MC80 with CNX and CRT supports this hypothesis, as both chaperones have been previously shown to bind and retain immature MHC-I in the ER [29,30,31]. Furthermore, four of the eight residues critical to sequence-independent association of MHC-I with peptides are not conserved in MC80, suggesting that MC80 may not bind peptides in an analogous manner. Potentially, the divergence of MC80 from MHC-I functions to mimic the peptide-receptive conformation of MHC-I to continually associate with CNX/PLC. As such, a structural analysis of MC80 may provide insight into the mechanism underpinning PLC-assisted peptide loading of classical MHC-I; particularly regarding the poorly understood interaction between MHC-I and Tpn.

Recent studies have made significant progress toward a structural understanding of MHC-I peptide loading by employing a protein with sequence similarity to Tpn, TAPBPR [40,67]. Like Tpn, TAPBPR is capable of peptide editing through association with MHC-I; however, its functional role in antigen presentation has not yet been fully resolved. It is interesting to note that, while expression of MC80 in Hela cells treated with IFNɣ dramatically decreased Tpn levels, TAPBPR levels remained unchanged compared to vector control. We hypothesize that this specificity is a result of the fact that E3 ligases usually conjugate ubiquitin with lysine residues [68]; whereas the cytoplasmic tail of Tpn has four lysines, the cytoplasmic tail of TAPBPR does not have any. However, it is possible that MC80 preferentially interacts with Tpn over TAPBPR; or that TAPBPR may be present in other cellular compartments where MC80 is absent. Regardless, MC80 expression appears to cause the specific degradation of Tpn and not TAPBPR.

While MC80 was originally predicted to be involved in NK-subversion, the mechanism described herein suggests that MC80 is involved in subverting CTL responses by downregulating MHC-I, which may in turn increase NK killing [69]. However, our data does not preclude MCV from encoding additional ORFs which subvert NK and CTL responses through distinct mechanisms. One such MCV protein, MC148, is known to function as an inhibitor of CCR8-mediated chemotaxis, limiting T cell migration into sites of infection [70]. MC80 likely works in concert with MC148 to prevent the activation of surveilling T cells, specifically those which have been able to localize to the MCV lesion. Comparatively, cowpox virus encodes at least seven distinct proteins suspected of antagonizing host chemokines [71], while also downregulating MHC-I expression by two independent mechanisms [72], and encoding at least one separate protein to prevent NK activation [24]. We therefore believe it unlikely that MC80 and MC148 make up the complete repertoire of immune evasion proteins that allow for apparent MCV subversion of both T and NK cell surveillance.

## Materials and methods

### Cell lines

Murine embryonic fibroblasts (MEF) cell lines including B6/WT3, TAP1-deficient (TAP1^-/-^; also referred to as FT1^-^), tapasin-deficient (Tpn^-/-^), triple knock-out (K^b-/-^, D^b-/-^, β2m^-/-^; 3KO), and L cells were gifts from Dr. Ted Hansen, and have been described previously [73]. Baf3 cells [74], a murine proB lymphocyte was obtained from Dr. Anthony French. The BV2 microglial cell line was a gift from Dr. Anthony Orvedahl, and have been described previously [75]. The murine T lymphocyte cell line RMA (ATCC: TIB-39), human embryonic kidney 293T cell line (HEK 293T; ATCC: CRL-3216), human cervical cancer cell line (Hela; ATCC: CCL-2), and human foreskin cell line (HFF-1; ATCC: SCRC-1041), were obtained from the American Type Culture Collection (ATCC, Manassas, VA). Hela cells used in this work were stably transfected with HLA-A2, indicated as Hela-A2. Cyt-SIINFEKL and ER-SIINFEKL MEFs were produced by stably transfecting a construct encoding SIINFEKL peptide conjugated to ubiquitin or a signal peptide, respectively. Atg5-KO BV2 cell line isolation was performed as described [76]. All cell lines were cultured in 5% CO_2_ at 37°C with RPMI-1640 (Gibco) supplemented with 10% fetal bovine serum (Gibco), 2mM L-glutamine, 10mM HEPES pH 7.2, 1mM sodium pyruvate, and 100U/mL penicillin/streptomycin. Where indicated, prior to harvesting for immunoprecipitation and immunoblotting, cells were cultured for 24–48 h with 100–150 units/mL of mouse or human interferon gamma (mIFNɣ, Invitrogen; hIFNɣ, R&D Systems), followed by an 8-9h incubation with 100nM Epoxomicin, 30μM MG132 (Calbiochem, MA) or 100μM chloroquine (Sigma). Cells were harvested and washed in PBS containing 20mM iodoacetamide twice before freezing cell pellets at -80°C for storage prior to processing. Where indicated dithiobis-succinimidyl propionate (DSP; Pierce) was added to wash buffer at a concentration of 2mM.

### Cloning of MC80 and retroviral transduction

The MC80 (MCV genotype 1) sequence was PCR amplified starting at M1 and M33 for constructs without the N-terminal Flag-tag or starting at Q18 and H72 for constructs with the N-terminal Flag-tag. Constructs with an N-terminal Flag-tag were inserted in frame with the canonical mouse β2m signal peptide and a Flag-tag into the pMXsIG vector (CellBioLabs) by overlap PCR and Gibson Assembly (NEB). In constructs that lacked an N-terminal Flag-tag, including the untagged construct and C-terminally Flag-tagged constructs, the native signal peptide was used for both the long and short forms of MC80. The soluble construct only included up to residue A342 of MC80, to truncate the predicted transmembrane and cytoplasmic tail. All constructs were confirmed by Sanger sequencing (GeneWiz). Retrovirus-containing supernatants were produced as per manufacturer instructions using either (i) the pVPack-GP and pVSVG vector (Agilent) in 293T cells to generate virus which infects human cell lines or (ii) the retroviral-packaging plat-E cells [77] to generate virus which infects murine cell lines. When necessary, retrovirally-transduced cells were enriched by cell sorting for GFP-positive cells (MoFlo).

### Antibodies

Rabbit anti-mouse TAP1 and ERp57, rabbit anti-human TAP1 and tapasin, and hamster anti-mouse tapasin (5D3) were gifts from Dr. Ted Hansen and have been described previously [33,35,78]. The rabbit anti-Derlin-1 antibody was a gift from Dr. Yihong Ye and has been described previously [62]. Anti-human tapasin (TO-3), anti-human TAPBPR (42-L), and anti-ubiquitin (P4D1) antibodies were purchase from Santa Cruz Biotechnology. Anti-β-actin (AC-74) and anti-Flag (M2); anti-GFP; rabbit anti-CRT and rabbit anti-calnexin; anti-CD1d and anti-Qa-1 were purchased from Sigma, Covance, Stressgen, and BD Pharmingen, respectively. Rae1a and MICA were detected using an NKG2D-tetramer (a gift from Dr. Sytse Piersma). All MHC-I mAbs including 11-4-1 (α-H-2K^k^), B8-24-3 (α-H-2K^b^), 30-5-7 (α-H-2L^d^), B22/249 (α-H-2D^b^), 25-D1-16 (α-H-2K^b^-SIINFEKL), BBM.1 (α-β_2_m), and W6/32 (HLA-ABC) were previously described and available from the ATCC collection.

### Flow cytometry

Staining was performed as described previously [79]. Phycoerythrin-conjugated goat anti-mouse IgG (BD Pharmingen) was used to visualize primary antibody staining. Intracellular staining was conducted using the BD cytofix/cytoperm kit (BD Pharmingen) following the manufacturer’s instructions. All flow cytometric analyses were performed using a FACSCalibur (Becton Dickinson). Data was analyzed using FlowJo 10 (Tree Star) and Prism 7 (GraphPad). Relative surface MHC-I expression % was calculated using the equation: [Mean fluorescence intensity (MFI) of MC80 positive population (GFP+)/MFI of MC80 negative population (GFP-)]*100. Error bars represent the standard deviation of 2 to 3 independent replicates.

### Immunoprecipitations and immunoblots

Cells were lysed in PBS buffer containing 20mM iodoacetamide, 1% IGEPAL CA-630 (Sigma), and cOmplete protease inhibitors (Roche). For coimmunoprecipitations, IGEPAL CA-630 was replaced with digitonin (Wako). After lysis for at least 30min on ice, homogenized lysates were incubated for 1hr with a saturating concentration of antibody that was either directly conjugated to resin or associated via resin-conjugated protein A. Beads were then washed four times with 0.1% IGEPAL-CA-630 or digitonin, and eluted with Flag peptide or LDS sample buffer (Invitrogen). If endoglycosidase H treatment followed the immunoprecipitation, bound proteins were instead eluted by boiling in 10mM TrisCl, pH 6.8, 0.5% SDS. Supernatants were then incubated with an equal volume of 100mM sodium acetate, pH 5.4, and 1–5 μU endoglycosidase H (NEB) for 1hr at 37°C. Immunoblotting was performed following SDS-PAGE separation of precipitated proteins or cell lysates as described previously [79]. Following primary blotting with mouse or hamster primary antibodies, membranes were blotted with biotin-conjugated goat anti-mouse IgG (Invitrogen) or goat anti-hamster IgG (Jackson ImmunoResearch), respectively, followed by blotting with streptavidin-horseradish peroxidase (Invitrogen). In cases where the biotin-conjugated anti-mouse system produced a high background, m-IgGk BP-HRP was substituted (Santa Cruz Biotechnology). For rabbit primary antibodies, HRP-conjugated mouse anti-rabbit IgG light chain (Jackson ImmunoResearch) was used instead. Specific proteins were visualized by chemiluminescence using ECL (Thermo).

### Statistics and bioinformatics

Statistical significance compared with the control group was calculated using ANOVA with Dunnett’s multiple comparisons test or unpaired t test and annotated as * = P<0.05; ** = P<0.01; *** = P<0.001; **** = P<0.0001. Protein alignments were conducted using the ESPRESSO webserver [80]. Signal peptide cleavage sites were predicted using the SignalP 4.1 webserver [81].

## Supporting information

S1 Fig MC80 encodes an ER-retained MHC-I-like protein. (A) A structure-informed sequence alignment of MC80 variants and HLA-A2 was performed using ESPRESSO, followed by minor manual adjustments. The start residue of the MC80L-F and MC80S-F constructs used in this work are labelled, as indicated by triangles. Constructs for F-MC80L and F-MC80S start with a β2m signal peptide and N-terminal Flag tag, and the start MC80 residues of these constructs are also labeled. Finally, the terminal residue of the soluble form of MC80 is also labeled. Regions of HLA-A2 with known functional roles are highlighted as: β2m-binding residues in blue, PLC-binding residues in yellow, peptide-backbone-associated residues in red, and multi-component-associated residues are shown in respective secondary colors. Solid colors indicate identical residues to the HLA-A2 sequence, and light colors indicate divergence. The HLA-A2 secondary structure is shown in gray above the sequences (α-helix: cylinder, β-sheet: arrow, transmembrane: box with lines). Conserved disulfide bonds are indicated with dashed lines. (B) Surface and intracellular 2D flow cytometry of HEK 293T cells transduced with vector control or N-terminally Flag-tagged MC80 constructs. While the bicistronically-expressed GFP serves as an indicator for retrovirally transduced cells, anti-Flag antibody specifically detects the surface or intracellular expression of MC80 protein. A representative plot of at least two independent replicates is shown. (C) HEK 293T and MEF-L^d^ cells expressing vector control or F-MC80S were immunoprecipitated with anti-FLAG antibody followed by an EndoH-sensitivity assay. Blots are representative of at least two independent experiments each.
(TIF)

S2 Fig MC80 downregulates MHC-I surface expression in (A) Hela-A2 cells and (B) Human foreskin fibroblasts (HFF-1). Cells were retrovirally transduced with the indicated MC80 constructs or vector control, followed by staining for HLA surface expression by a pan-MHC-I (W6/32) or HLA-A2-specific (BB7.2) antibody. The mean fluorescence intensity (MFI) is indicated for GFP+ and GFP- cells in each flow cytometry experiment. Plots are representative of (A) two independent experiments and (B) one experiment run in duplicate.
(TIF)

S3 Fig Quantified EndoH-sensitivity of L^d^ by MC80 and downregulation of MHC-I by CPXV012 and CPXV203 in the presence of SIINFEKL. (A) Fraction of mature L^d^ (EndoH-resistant) in the presence or absence of MC80, as also depicted in Fig 3A, is quantified. Error bars represent the standard deviation of two independent replicates. (B) SIINFEKL was expressed in MEFs using a retroviral transduction system, as shown in Fig 3B. The relative MHC-I level of GFP+ / GFP- cells is indicated as a percentage for each plot. CPXV012 inhibits TAP-mediated peptide transport, and therefore only downregulates MHC-I when SIINFEKL is expressed in the cytosol. CPXV203 directly binds mature MHC-I, retaining it in the ER, and therefore downregulates MHC-I independent upon the localization of SIINFEKL-expression. Representative plots of two independent experiments are shown.
(TIF)

S4 Fig Immunoprecipitations of MC80 with MHCI-related proteins reveal that tapasin degradation by MC80 can prevent identification of the association between MC80 and tapasin, depending on the cell line/treatment. (A) HEK-293T cells, (B) untreated MEFs (Tpn -/- and TAP1 -/-), and (C) MEF-L^d^ cells were retrovirally transduced with MC80 constructs or vector control. (A) HEK293T cell lysates were immunoprecipitated by FLAG. Elutants were blotted for FLAG, Tpn, TAP1, and lysate was blotted for actin as a control. The soluble form of MC80 was found to associate with both Tpn and TAP1, while the association with Tpn was not detectable in functional forms of MC80. HEK 293T FLAG-IPs and blots are representative of two independent experiments, once with DSP-crosslinking and once without. Blots from the DSP-crosslinked experiment are shown. (B) FLAG-IPs of untreated Tpn/TAP-deficient MEFs were blotted for FLAG, CNX, CRT, TAP, and Tpn. (C) MEF-L^d^ cell lysates were immunoprecipitated for FLAG. Elutants were blotted for FLAG and β_2_m and lysates were blotted for actin as a loading control. Representative blots of two independent experiments are shown. Ladder markers for western blots indicate the protein mass in kilodaltons.
(TIF)

S5 Fig MC80-mediated degradation of Tpn is specifically proteasome-dependent. (A) Representative data/blots from the experiment depicted in Fig 7A. MEF-L^d^ cells retrovirally transduced with vector control of MC80L-F were treated with mIFNɣ for 24 hours and with MG132, DMSO control, Chloroquine, or PBS control for 9 hours. Lysates were blotted for actin and Tpn with either a high or low sample load onto the SDS-PAGE gel. Low sample loads were used for final quantification (S5A Fig right panel; Fig 7A). (B) Atg5 KO BV2 microglial cell lines, with and without Atg5 trans-complemented, were retrovirally transduced with vector control of MC80L-F, followed by staining for D^b^ or K^b^ and quantification by flow cytometry. Error bars represent the standard deviation of two independent replicates. (C) Representative data/blots from the experiment in Fig 7D are shown. Hela-A2 cells retrovirally transduced with vector control or MC80S-F were treated with hIFNɣ followed by Epoxomicin or DMSO control for 9 hours. Lysates were blotted for actin and Tpn and respective Tpn levels were quantified (S5C Fig right panel; Fig 7D). Error bars are representative of two independent experiments. Ladder markers for western blots indicate the protein mass in kilodaltons.
(TIF)
