# Supplementary figures and images for: Molluscum contagiosum virus MC80 sabotages MHC-I antigen presentation by targeting tapasin for ER-associated degradation
Source: PLoS Pathog. 2019 Apr 29;15(4):e1007711. doi: 10.1371/journal.ppat.1007711 (PMC6508746; doi:10.1371/journal.ppat.1007711)

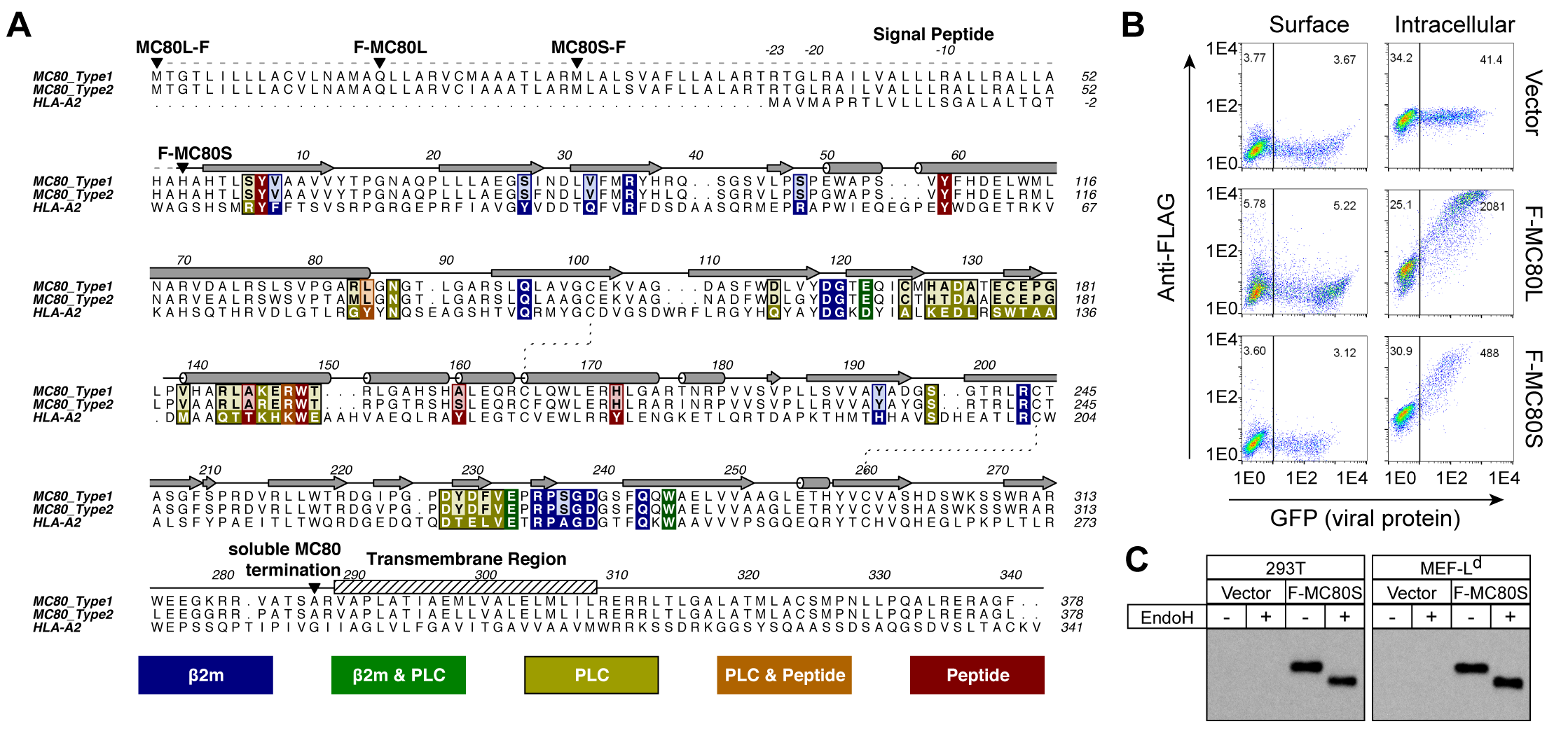

Supplement: S1 Fig — (A) A structure-informed sequence alignment of MC80 variants and HLA-A2 was performed using ESPRESSO, followed by minor manual adjustments. The start residue of the MC80L-F and MC80S-F constructs used in this work are labelled, as indicated by triangles. Constructs for F-MC80L and F-MC80S start with a β2m signal peptide and N-terminal Flag tag, and the start MC80 residues of these constructs are also labeled. Finally, the terminal residue of the soluble form of MC80 is also labeled. Regions of HLA-A2 with known functional roles are highlighted as: β2m-binding residues in blue, PLC-binding residues in yellow, peptide-backbone-associated residues in red, and multi-component-associated residues are shown in respective secondary colors. Solid colors indicate identical residues to the HLA-A2 sequence, and light colors indicate divergence. The HLA-A2 secondary structure is shown in gray above the sequences (α-helix: cylinder, β-sheet: arrow, transmembrane: box with lines). Conserved disulfide bonds are indicated with dashed lines. (B) Surface and intracellular 2D flow cytometry of HEK 293T cells transduced with vector control or N-terminally Flag-tagged MC80 constructs. While the bicistronically-expressed GFP serves as an indicator for retrovirally transduced cells, anti-Flag antibody specifically detects the surface or intracellular expression of MC80 protein. A representative plot of at least two independent replicates is shown. (C) HEK 293T and MEF-Ld cells expressing vector control or F-MC80S were immunoprecipitated with anti-FLAG antibody followed by an EndoH-sensitivity assay. Blots are representative of at least two independent experiments each. (TIF) [file ppat.1007711.s001.tif]

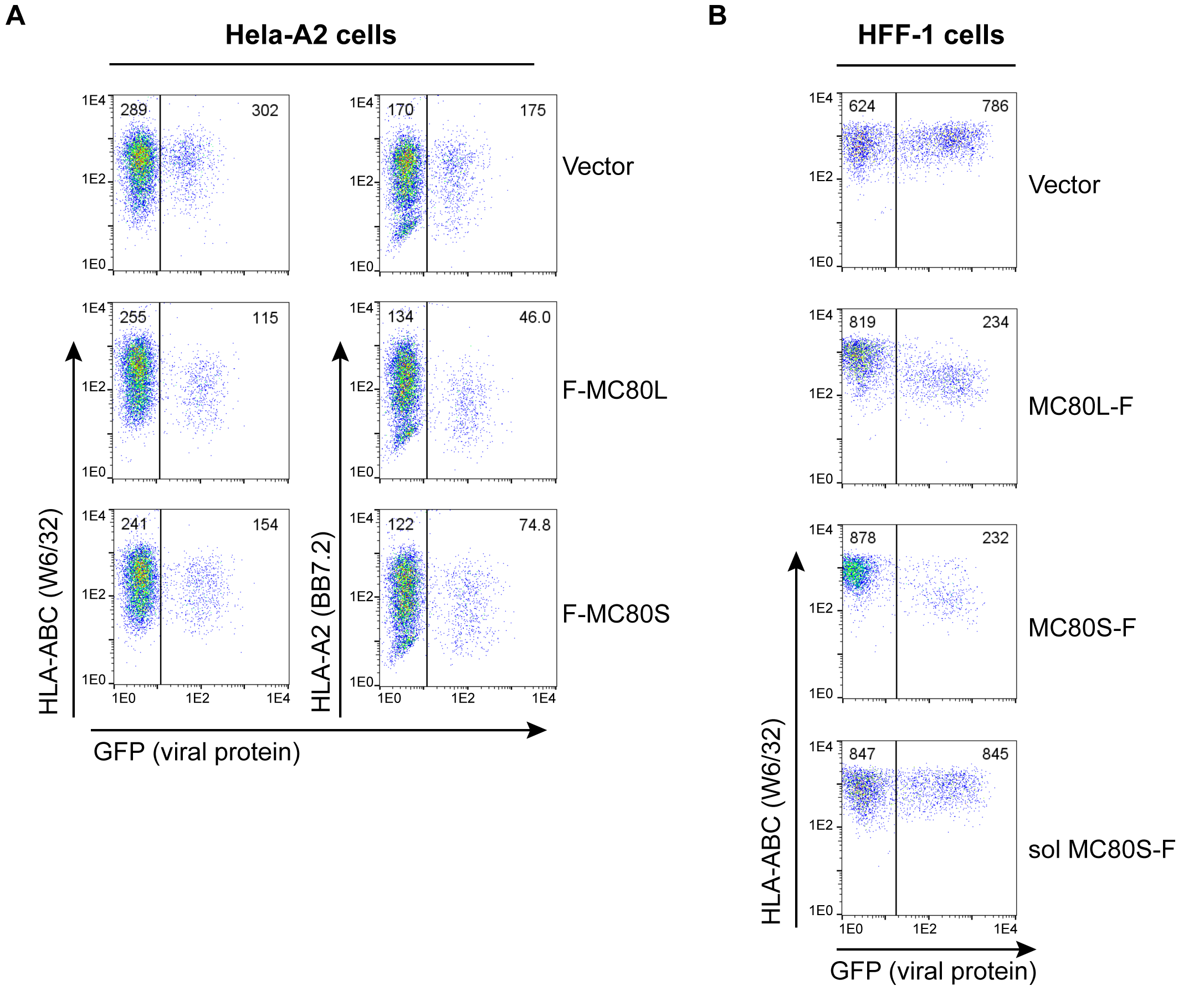

Supplement: S2 Fig — Cells were retrovirally transduced with the indicated MC80 constructs or vector control, followed by staining for HLA surface expression by a pan-MHC-I (W6/32) or HLA-A2-specific (BB7.2) antibody. The mean fluorescence intensity (MFI) is indicated for GFP+ and GFP- cells in each flow cytometry experiment. Plots are representative of (A) two independent experiments and (B) one experiment run in duplicate. (TIF) [file ppat.1007711.s002.tif]

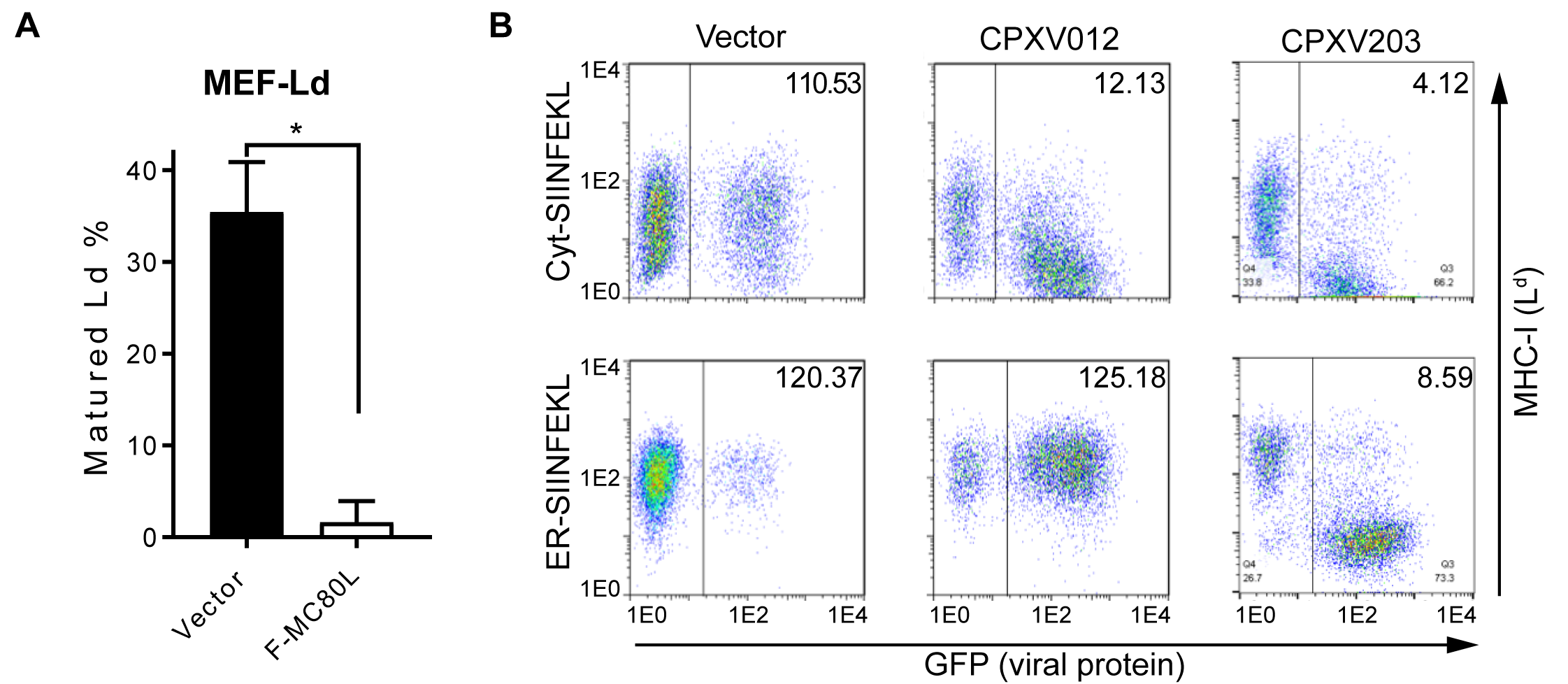

Supplement: S3 Fig — (A) Fraction of mature Ld (EndoH-resistant) in the presence or absence of MC80, as also depicted in Fig 3A, is quantified. Error bars represent the standard deviation of two independent replicates. (B) SIINFEKL was expressed in MEFs using a retroviral transduction system, as shown in Fig 3B. The relative MHC-I level of GFP+ / GFP- cells is indicated as a percentage for each plot. CPXV012 inhibits TAP-mediated peptide transport, and therefore only downregulates MHC-I when SIINFEKL is expressed in the cytosol. CPXV203 directly binds mature MHC-I, retaining it in the ER, and therefore downregulates MHC-I independent upon the localization of SIINFEKL-expression. Representative plots of two independent experiments are shown. (TIF) [file ppat.1007711.s003.tif]

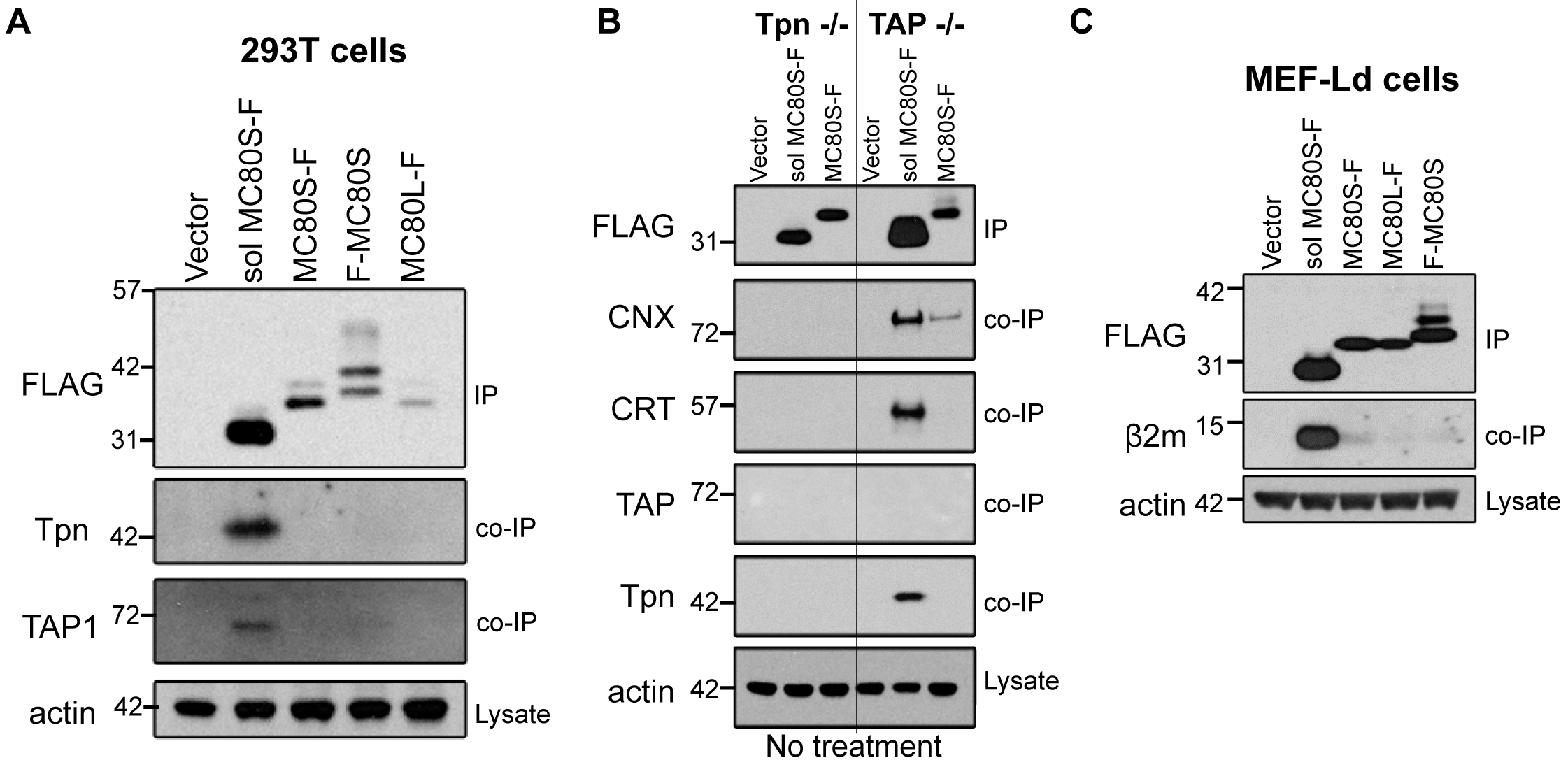

Supplement: S4 Fig — (A) HEK-293T cells, (B) untreated MEFs (Tpn -/- and TAP1 -/-), and (C) MEF-Ld cells were retrovirally transduced with MC80 constructs or vector control. (A) HEK293T cell lysates were immunoprecipitated by FLAG. Elutants were blotted for FLAG, Tpn, TAP1, and lysate was blotted for actin as a control. The soluble form of MC80 was found to associate with both Tpn and TAP1, while the association with Tpn was not detectable in functional forms of MC80. HEK 293T FLAG-IPs and blots are representative of two independent experiments, once with DSP-crosslinking and once without. Blots from the DSP-crosslinked experiment are shown. (B) FLAG-IPs of untreated Tpn/TAP-deficient MEFs were blotted for FLAG, CNX, CRT, TAP, and Tpn. (C) MEF-Ld cell lysates were immunoprecipitated for FLAG. Elutants were blotted for FLAG and β2m and lysates were blotted for actin as a loading control. Representative blots of two independent experiments are shown. Ladder markers for western blots indicate the protein mass in kilodaltons. (TIF) [file ppat.1007711.s004.tif]

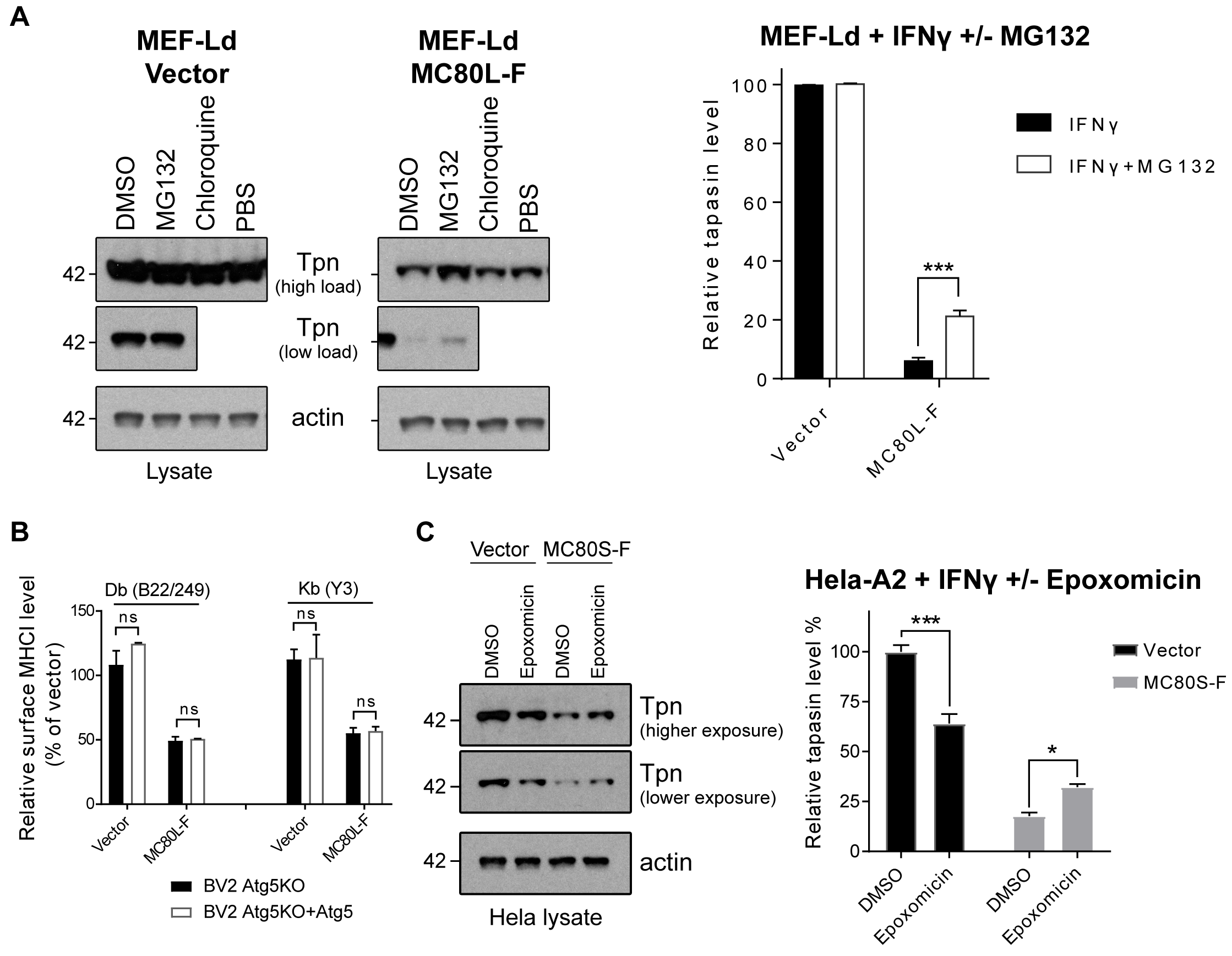

Supplement: S5 Fig — (A) Representative data/blots from the experiment depicted in Fig 7A. MEF-Ld cells retrovirally transduced with vector control of MC80L-F were treated with mIFNɣ for 24 hours and with MG132, DMSO control, Chloroquine, or PBS control for 9 hours. Lysates were blotted for actin and Tpn with either a high or low sample load onto the SDS-PAGE gel. Low sample loads were used for final quantification (S5A Fig right panel; Fig 7A). (B) Atg5 KO BV2 microglial cell lines, with and without Atg5 trans-complemented, were retrovirally transduced with vector control of MC80L-F, followed by staining for Db or Kb and quantification by flow cytometry. Error bars represent the standard deviation of two independent replicates. (C) Representative data/blots from the experiment in Fig 7D are shown. Hela-A2 cells retrovirally transduced with vector control or MC80S-F were treated with hIFNɣ followed by Epoxomicin or DMSO control for 9 hours. Lysates were blotted for actin and Tpn and respective Tpn levels were quantified (S5C Fig right panel; Fig 7D). Error bars are representative of two independent experiments. Ladder markers for western blots indicate the protein mass in kilodaltons. (TIF) [file ppat.1007711.s005.tif]
